# Genetic Interaction Among Phytochrome, Ethylene and Abscisic Acid Signaling During Dark-Induced Senescence in *Arabidopsis thaliana*

**DOI:** 10.3389/fpls.2020.00564

**Published:** 2020-05-21

**Authors:** Hiroaki Ueda, Takeshi Ito, Ryouhei Inoue, Yu Masuda, Yumi Nagashima, Toshiaki Kozuka, Makoto Kusaba

**Affiliations:** Graduate School of Integral Sciences for Life, Hiroshima University, Higashihiroshima, Japan

**Keywords:** senescence, ethylene, ABA, strigolactone, phytochrome-interacting factor, ORE1, AtNAP, sugar

## Abstract

Leaf senescence is induced by various internal and external stimuli. Dark-induced senescence has been extensively investigated, but the detailed mechanism underlying it is not well understood. The red light/far-red light receptor phytochrome B and its downstream transcription factors, PYHTOCHROME INTERACTING FACTORs (PIFs) 4 and 5, are known to play an important role in dark-induced senescence. Furthermore, the senescence-inducing phytohormones, ethylene and abscisic acid (ABA) are reported to be involved in dark-induced senescence. In this study, we analyzed the relationship between ethylene, ABA and PIFs in dark-induced leaf senescence. A triple mutant of the core ABA signaling components; SNF1-related protein kinases 2D (SRK2D), SRK2E, and SRK2I, displayed an ABA insensitive phenotype in ABA-induced senescence, whilst the ethylene insensitive mutant *ein2* demonstrated low sensitivity to ABA, suggesting that ethylene signaling is involved in ABA-induced senescence. However, the *pif4 pif5* mutant did not display low sensitivity to ABA, suggesting that PIF4 and PIF5 act upstream of ABA signaling. Although PIF4 and PIF5 reportedly regulate ethylene production, the triple mutant *ein2 pif4 pif5* showed a stronger delayed senescence phenotype than *ein2* or *pif4 pif5*, suggesting that EIN2 and PIF4/PIF5 partially regulate leaf senescence independently of each other. While direct target genes for PIF4 and PIF5, such as *LONG HYPOCOTYL IN FAR-RED1* (*HFR1*) and *PHYTOCHROME INTERACTING FACTOR 3-LIKE 1* (*PIL1*), showed transient upregulation under dark conditions (as is seen in the shade avoidance response), expression of *STAY GREEN1* (*SGR1*), *ORESARA1* (*ORE1*) and other direct target genes of PIF5, continued to increase during dark incubation. It is possible that transcription factors other than PIF4 and PIF5 are involved in the upregulation of *SGR1* and *ORE1* at a later stage of dark-induced senescence. Possible candidates are senescence-induced senescence regulators (SIRs), which include the NAC transcription factors ORE1 and AtNAP. In fact, ORE1 is known to bind to the *SGR1* promoter and promotes its expression. It is therefore inferred that the phytochrome-PIF pathway regulates initial activation of senescence and subsequently, induced SIRs reinforce leaf senescence during dark-induced senescence.

## Introduction

Leaf senescence is a system to recover nutrients from unnecessary leaves, which is accompanied by drastic changes in metabolism, gene expression and cell structure. Leaf senescence is induced, not only by internal factors such as aging and flowering, but also by various external stimuli such as prolonged dark incubation, drought, and salinity (Schippers, 2015). However, common phenomena such as yellowing (chlorophyll degradation) and reduction in photosynthetic activity, known as “senescence syndrome” occur irrespective of stimuli that cause leaf senescence (Noodén, 2004).

Leaf senescence is regulated by several phytohormones (Kusaba et al., 2013). For example, ethylene, which is well known to be an important senescence-inducing phytohormone (Grbić and Bleecker, 1995; Oh et al., 1997). Impairment of ethylene signaling components, such as ETHYLENE INSENSITIVE2 (EIN2) and EIN3/EIN3 LIKE1 (EIL1), severely represses leaf senescence in dark-induced senescence. Mutants of strigolactone synthesis genes, such as *MORE AXILLARY GROWTH1* (*MAX1*), *MAX3*, and *MAX4* and the signaling genes *ARABIDOPSIS DOWARF 14* (*AtD14*) and *MAX2*, also exhibit a delayed senescence phenotype, suggesting that strigolactone is a senescence-promoting phytohormone (Woo et al., 2001; Ueda and Kusaba, 2015). Abscisic acid (ABA) also induces leaf senescence, and overexpression of its receptor genes *PYR1-LIKE 8* (*PYL8*) and *PYL9* confers an early senescence phenotype (Lee et al., 2015; Zhao et al., 2016). In addition, the ABA signaling transcription factors ABA INSENSITIVE5 (ABI5) and ENHANCED EM LEVEL (EEL) are reportedly involved in leaf senescence (Sakuraba et al., 2014). Further phytohormones such as jasmonic acid (Zhuo et al., 2020) and salicylic acid (Yoshimoto et al., 2009) are also known to be involved in leaf senescence. These phytohormones are thought to function downstream of senescence-inducing stimuli. The examination of crosstalk between phytohormones is important in order to clarify the complete picture of regulation of leaf senescence in plants. For example, the senescence promoting function of strigolactone is strongly associated with ethylene signaling (Ueda and Kusaba, 2015).

Among several leaf senescence-inducing stimuli, prolonged dark incubation has been the most extensively studied (Liebsch and Keech, 2016). Under dark conditions, limitation of photosynthesis causes sugar starvation in the plant. Reportedly, sugar plays an important role in natural leaf senescence, but its role in dark-induced senescence remains unclear (Moore et al., 2003; Pourtau et al., 2006). On the other hand, it is well established that dark-induced senescence is regulated by the core components of the shade avoidance response, which allows plants to escape from shade in order to acquire light energy (Whitelam and Smith, 1991; Franklin and Whitelam, 2005). The red/far-red light receptor phytochrome degrades its downstream bHLH transcription factors; PHYTOCHROME INTERACTING FACTORs (PIFs) to repress the shade avoidance response (Quail et al., 1995; Legris et al., 2019). *HFR1*, *PIL1*, and *ARABIDOPSIS THALIANA HOMEOBOX PROTEIN2* (*AtHB2*) are known to be the direct targets of PIFs and function in shade avoidance responses. Since mutants of PIF4 and PIF5, which are degraded by Phytochrome B (phyB) (activated by red light), show a strong delayed senescence phenotype in the dark-induced senescence, PIF4 and PIF5 are considered as the central regulators of dark-induced senescence (Sakuraba et al., 2014; Song et al., 2014; Zhang et al., 2015).

During dark-induced senescence, ethylene production is increased, but has been found to be reduced in *pif4*, suggesting that ethylene production is regulated by the phyB-PIF pathway (Song et al., 2014; Ueda and Kusaba, 2015). Consistent with this, the expression of several ACC synthase genes, which are the rate-limiting enzymes in ethylene production, is upregulated during dark incubation, but this upregulation is repressed in *pif* mutants (Song et al., 2014; Qiu et al., 2015). Furthermore, PIF5 directly regulates transcription of the master transcription factor of ethylene signaling, EIN3, suggesting that PIFs regulate ethylene signaling (Sakuraba et al., 2014).

In addition to PIFs and EIN3, NAC and WRKY transcription factors are known to be involved in dark-induced senescence (Kusaba et al., 2013; Kim et al., 2016). Among such NAC transcription factors, ORE1 has been the most intensely analyzed. *ORE1* is induced during leaf senescence and promotes leaf senescence (Kim et al., 2009). EIN3 and PIF4/PIF5 directly regulate expression of *ORE1*, and at the same time, EIN3 represses expression of *miR164* targeting *ORE1* (Li et al., 2013; Sakuraba et al., 2014; Qiu et al., 2015). ORE1 is also involved in a wide spectrum of leaf senescence, including aging (Kim et al., 2009), reactive oxygen species (Woo et al., 2004), ethylene (Kim et al., 2009, 2014), jasmonic acid (Kim et al., 2014), salinity (Balazadeh et al., 2010), the circadian clock (Kim et al., 2018) and nitrogen starvation (Park et al., 2018). Another senescence-induced NAC transcription factor, AtNAP, is also known to be involved in natural senescence (Guo and Gan, 2006) and drought (Zhang and Gan, 2012; Sakuraba et al., 2015) and dark-induced senescence (Yang et al., 2014). In addition, a class of NAC transcription factors, sNACs, are reportedly required for ABA-induced senescence (Takasaki et al., 2015). Furthermore, VND-INTERACTING2 (VNI2) and JUNGBRUNNEN1 (JUB1) are also senescence-induced NAC transcription factors, however, they represse the progression of leaf senescence (Yang et al., 2011; Wu et al., 2012). Since these NAC transcription factor genes are induced during leaf senescence and regulate leaf senescence, they can be referred to as senescence-induced senescenceregulators (SIRs). Senescence-regulating WRKY genes such as WRKY53 are also thought to belong to the SIRs group (Hinderhofer and Zentgraf, 2001).

Senescence syndrome is eventually caused by senescence-related enzymes, which is generally induced or activated during leaf senescence. Such enzymes include the chlorophyll-degrading enzymes SGR/NON-YELLOWING1 (NYE1), NON-YELLOW COLORING1 (NYC1), NYC1 LIKE (NOL) and PHEOPHYTINASE (PPH) /NYC3 (Kusaba et al., 2007; Ren et al., 2007; Sato et al., 2007, 2009; Horie et al., 2009; Morita et al., 2009; Schelbert et al., 2009; Shimoda et al., 2016), the cysteine protease SENESCENCE ASSOCIATED GENE12 (SAG12) (Weaver et al., 1998), and the organelle endonuclease DEFECTIVE IN POLLEN ORGANELLE DNA DEGRADATION1 (DPD1) (Takami et al., 2018). These enzymes are thought to execute cellular senescence. Such senescence-induced genes can be referred to as senescence-induced senescence executers (SIEs), whose expression may be controlled by senescence-regulating transcription factors.

Thus, leaf senescence progresses through complex interactions between senescence-related factors. The molecular basis of leaf senescence is not yet completely understood. Such processes have been analyzed utilizing delayed/early senescence mutants. However, there are few reports that examine the genetic interaction of these mutants. In this study, we genetically and physiologically dissect the roles of senescence-inducing-pathways in dark-induced leaf senescence using several delayed senescence mutants and their multiple mutants to better understand the complete picture of regulation of dark-induced leaf senescence in plants.

## Materials and Methods

### Plant Material and Growth Conditions

The *Arabidopsis thaliana* ecotype Columbia (Col-0) was used as the wild type plant. The mutant lines *pif4-102* (SALK_140393; Sun et al., 2013), *pif5-3* (SALK_087012; Sun et al., 2013), *ein2-5* (CS16771; Alonso et al., 1999), *ore1-3* (SALK_090154; Kim et al., 2014), and *atnap* (SALK_005010; Guo and Gan, 2006) were obtained from the Arabidopsis Biological Resource Center (ABRC). *pif4 pif 5* double mutant was generated by a cross between *pif4-102* and *pif5-3*. The *srk2d/e/i* triple mutant (Fujita et al., 2009; Umezawa et al., 2009) was kindly provided by Kazuo Shinozaki (RIKEN Center for Sustainable Resource Science). Typically, plants were grown on soil in a growth chamber for a 4-week period under the following conditions: 22°C, 10 h light/14 h dark (short-day) photoperiod, and 80 μmol⋅photons m^–2^⋅s^–1^. For the natural senescence experiments, plants were grown under long-day conditions (16 h light/8 h dark).

### Dark and Phytohormone Treatments

For the dark treatment, the 7th or 8th leaves from the top of 4-week-old plants with 16 leaves were detached and incubated in a box with constant high humidity, in the dark at 22°C. For the light (control) and phytohormone treatments, leaves were incubated under continuous white light conditions (5 μmol m^–2^ s^–1^) at 22°C. For the physiological treatment, leaves were incubated in 0.5% (w/v) agar media containing 3 mM 2-(*N*-morpholino) ethanesulfonic acid (MES, pH 5.8) supplemented with abscisic acid (Sigma-Aldrich; St. Louis, MO, United States), glucose, or mannitol.

### Measurement of Senescence Parameters

Chlorophyll content was measured using a SPAD-502 chlorophyll meter as SPAD value (Konica-Minolta, Japan). Average of SPAD values of three positions in a detached leaf were used. *F_v_/F_m_* values were measured using a Junior PAM chlorophyll fluorometer (Walz; Germany) according to Kohzuma et al. (2017). Detached leaves kept in dark for 30 min were subjected to measurement of *F_v_/F_m_*.

### RNA Extraction and qRT-PCR Analysis

Total RNA was extracted using an Isospin with a spin column (Nippon Gene, Japan). First-strand cDNA was synthesized from 100 ng total RNA using ReverTra Ace qPCR RT Master Mix (Toyobo, Japan). Quantitative, reverse-transcriptase polymerase chain reaction (qRT-PCR) was performed using a KAPA SYBR FAST qPCR kit (Nippon Genetics; Tokyo, Japan) and a Roter-Gene Q 2PLEX (Qiagen; Venlo, Netherlands). qRT-PCR conditions are as follows: initial denaturation at 94°C for 3 min, followed by 40 cycles at 95°C for 5 s, and then 60°C for 10 s. The transcript level of each gene was normalized to that of *ACTIN8* (*ACT8*). Data analysis was performed using the Comparative CT Method, ΔΔCT Method (Livak and Schmittgen, 2001). The sequences of primers used for qRT-PCR are shown in Supplementary Table S1.

### Accession Numbers

Sequence data from this article can be found in the Arabidopsis Genome Initiative under the following accession numbers: *PIF4*(AT2G43010), *PIF5*(AT3G59060), *HFR1*(AT1G02340), *PIL1*(AT2G46970), *AtHB2*(AT4G16780), *EIN2* (At5g03280), *SRK2D*(AT3G50500), *SRK2E*(AT4G33950), *SRK2I*(AT5G66880), *ORE1* (At5g39610), *MAX4*(AT4G32810), *SGR1*(At4 g22920), *SAG12* (At5g45890), *AtNAP* (At1g69490), and *ACT8*(AT1G49240).

## Results

### Effect of Sugar on Dark-Induced Senescence

In the majority of plant species, prolonged dark treatment induces leaf senescence. Under dark conditions, photosynthesis is not performed, resulting in sugar starvation. To investigate the role of sugar starvation on dark-induced senescence, we analyzed leaf senescence during dark incubation in the presence of sugar (Supplementary Figure S1). Leaves that were the 6th from the top were selected from 4-week-old *A. thaliana* plants and incubated in mock medium or media containing either 50 mM glucose or 50 mM mannitol under dark conditions. Almost no reduction in chlorophyll contents and *Fv/Fm* values, which reflect photosystem II activity, was observed under light conditions but chlorophyll was severely degraded during dark incubation (Supplementary Figures S1A,B). Degrees of reduction of chlorophyll contents and *Fv/Fm* values were similar across dark-incubated mock medium, 50 mM glucose, and 50 mM mannitol-treated leaves during dark incubation. However, the chlorophyll content of 50 mM glucose-treated leaves 3 days after the start of treatment (DAT) and the *Fv/Fm* value of 50 mM mannitol-treated leaves at 5 DAT were slightly higher than those of the dark-treated mock condition (Supplementary Figure S1A,B). Expression of *DIN6*, a marker gene for sugar starvation (Baena-González et al., 2007), was drastically upregulated in the dark-treated mock condition, but this upregulation was repressed by 50 mM glucose treatment, suggesting that repression of sugar starvation could not fully repress dark-induced leaf senescence. In addition, the 50 mM mannitol treatment, which does not fully repress sugar starvation, evoked similar responses to simple dark treatment, confirming that sugar starvation is not the major cause of dark-induced senescence.

### PIF4/PIF5- Dependent Gene Expression in Dark-Induced Senescence

**FIGURE 1:**
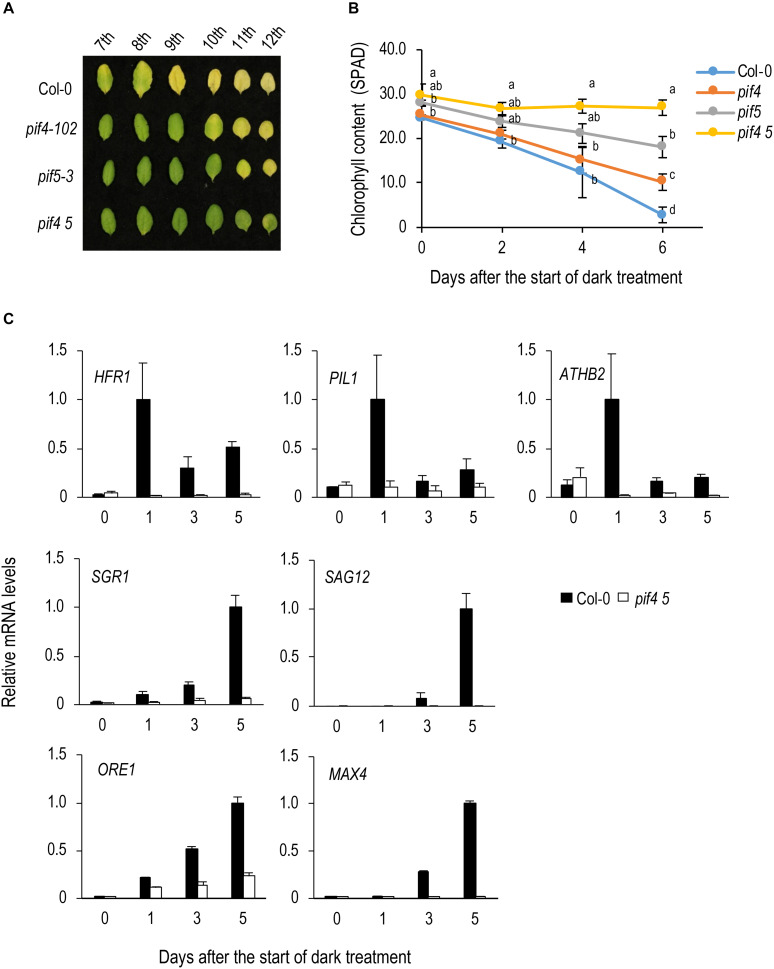
Analysis of the function of PIF4 and PIF5 in dark-induced senescence. **(A)** Dark-induced leaf senescence in *pif4⋅pif5*. Leaves in the 7th to 12th positions from the top of 4-week-old plants were incubated in the dark for 6 days. **(B)** Changes in chlorophyll content over time in *pif4 pif5* during dark incubation. Detached 8th leaves were used for the analysis. Data for the same day after the start of treatment were statistically compared using Tukey’s multiple comparison method (*p* < 0.05). **(C)** qRT-PCR analysis of transcript levels of direct target genes of PIF5 and senescence-induced genes. 8th leaves were used for the analysis. Solid and open bars indicate Col-0 and *pif4 pif5* leaves, respectively. Transcript levels were relative to that in Col-0 leaves at 5 DAT. *ACT8* was used as a reference. Bars indicate standard error (*n* = 3).

Reportedly, PIF4 and PIF5 play an important role in dark-induced leaf senescence in *A. thaliana*. To confirm this, we detached and incubated leaves from between the 7th to 12th position from the top of 4-week-old plants in the dark. At 6 DAT, upper (7th to 9th) leaves of Col-0 turned yellow but those of *pif4-102* and *pif5-3* remained green, and in *pif4 pif5* even lower (10th to 12th) leaves remained green (Figures 1A,B), confirming that *pif4* and *pif5* are delayed senescence mutants and *pif4 pif5* shows a stronger phenotype.

PIF4 and PIF5 play an important role in shade avoidance and their direct target genes, such as *HFR1*, *PIL1* and *AtHB2* have been identified (Kunihiro et al., 2011; Hornitschek et al., 2012). We examined the expression of these genes in Col-0 leaves during dark incubation and found that expressions of all of them are transiently upregulated at 1 DAT (Figure 1C). Such behavior is similar to that observed in high red light/far red light (R/FR) to low R/FR shift in young seedlings, which is thought to reflect a negative feedback regulation of shade avoidance (Sessa et al., 2005; Hornitschek et al., 2012). In *pif4 pif5*, such upregulation of *HFR1*, *PIL1*, and *AtHB2* is not observed, confirming that they are also regulated by PIF4 and PIF5 in dark-treated leaves (Figure 1C). Expression of the senescence-induced genes, *SGR1*, *SAG12*, *ORE1*, and MAX4, is severely repressed in *pif4 pif5*, confirming that they are regulated by PIF4 and PIF5 (Figure 1C). Among them, *ORE1* and *SGR1* are thought to be direct targets of PIF4/PIF5 (Sakuraba et al., 2014; Song et al., 2014; Zhang et al., 2015). However, *ORE1* and *SGR1* demonstrated a continuous increase in expression rather than the transient upregulation observed in *HFR1*, *PIL1* and *AtHB2*. Meanwhile, the expression of *PIF4* and *PIF5* was not upregulated during dark incubation (Supplementary Figure S2).

### Roles of ABA in Dark-Induced Senescence

**FIGURE 2:**
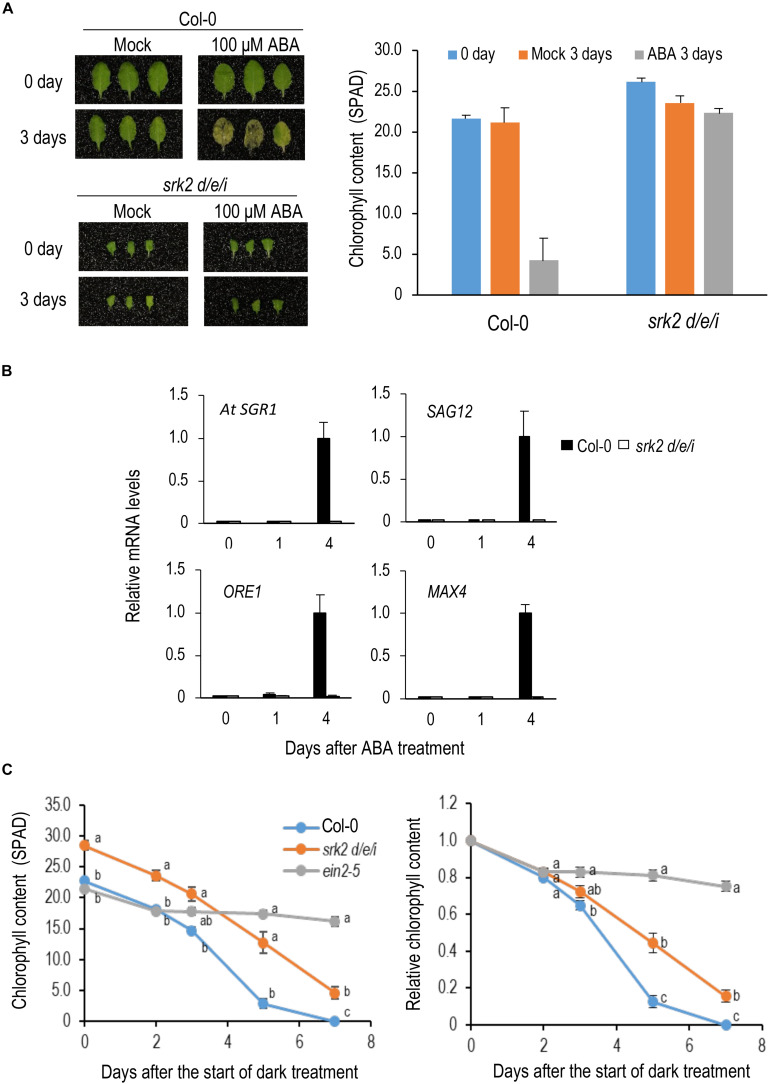
Roles of ABA in dark-induced leaf senescence. **(A)** Sensitivity of *srk2d/e/i* to ABA in leaf senescence. Left, leaves in the 8th position from the top of 4-week-old plants were incubated on the medium containing 100 μM ABA for 3 days under light conditions. Right, Changes in chlorophyll content by ABA treatment. **(B)** qRT-PCR analysis of changes in transcript levels of senescence-induced genes during ABA treatment. Detached 8th position leaves were used for the analysis. *ACT8* was used as a reference. Solid and open bars indicate Col-0 and *srk2d/e/i*, respectively. Transcript levels were relative to that in Col-0 leaves 4 days after ABA treatment. *ACT8* was used as a reference. **(C)** Changes in chlorophyll content over time in *srk2d/e/i* during dark incubation. Detached 8th position leaves were used for the analysis. Chlorophyll content is indicated in SPAD value in the left panel and those relative to day 0 are shown in the right panel. Data for the same day after the start of dark incubation were statistically compared using Tukey’s multiple comparison method (*p* < 0.05). Bars indicate standard errors (*n* = 6, 4, and 8 in **A**, **B**, and **C**, respectively).

Although ABA is known as a phytohormone that promotes leaf senescence, its genetic contribution to dark-induced senescence has not been examined in detail. To understand its contribution to dark-induced senescence, we analyzed the triple mutant of *SRK2D/E/I*, which encode protein kinases that play a central role in core ABA signaling (Fujii and Zhu, 2009; Fujita et al., 2009). First, we examined the effect of ABA on leaf senescence under light conditions. The 8th leaves from the top of 4-week-old Col-0 and *srk2d/e/i* plants were treated with 100 μM ABA. Col-0 leaves turned yellow but *srk2d/e/i* did not show any yellowing at 3 DAT (Figure 2A). Expression of senescence-induced genes was upregulated in Col-0 but not in *srk2d/e/i* at 3 DAT (Figure 2B), suggesting that *srk2d/e/i* is insensitive to ABA in leaf senescence.

Next, we incubated *srk2d/e/i* leaves in the dark. *srk2d/e/i* also showed delayed senescence phenotype during dark incubation, but it was much weaker than the ethylene-insensitive mutant *ein2-5*, suggesting that its contribution of ABA to dark-induced senescence is limited (Figure 2C).

### Crosstalk Between ABA and Ethylene in Leaf Senescence

**FIGURE 3:**
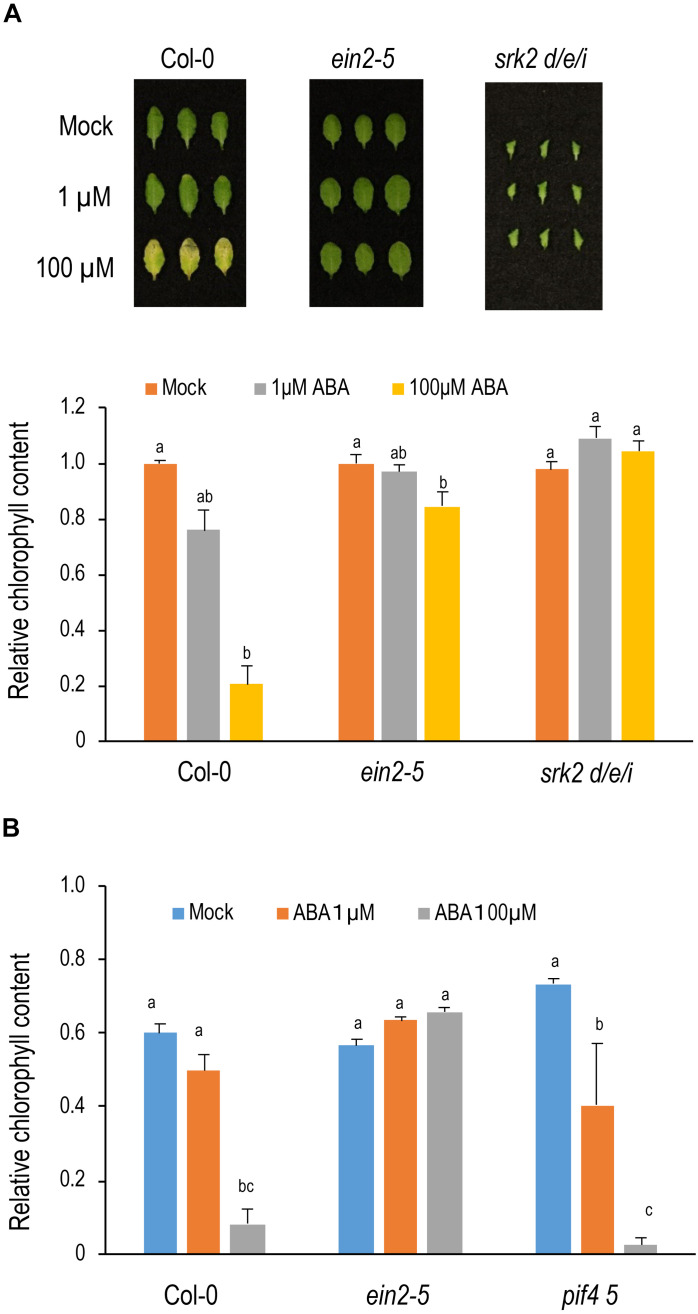
Sensitivity of *ein2*and *pif4 pif5* to ABA in leaf senescence. **(A)** Sensitivity of *ein2-5* and *srk2d/e/i* to ABA in leaf senescence under light conditions. Leaves positioned 8th from the top of 4-week-old plants were incubated on the medium containing 1 or 100 μM ABA for 3 days under light conditions. Lower panel shows chlorophyll content (SPAD value) relative to those of mock condition in each genotype. **(B)** Sensitivity of *ein2-5* and *pif4 pif5* to ABA in leaf senescence under dark conditions. The 8th leaves were incubated on the medium containing 1 or 100 μM ABA for 3 days under dark conditions. Chlorophyll content (SPAD value) were relative to those before ABA treatment in each genotype. Data were statistically compared using Tukey’s multiple comparison method in each genotype in A and in all genotypes in B (*p* < 0.05). Bars indicate standard errors (*n* = 8 in **A**, and *n* = 4 in **B**).

To investigate the crosstalk between ABA and ethylene, *ein2-5* was treated with 1 μM or 100 μM ABA for 3 days under light conditions. Chlorophyll content was slightly reduced under 1 μM ABA and leaves turned yellow under 100 μM ABA in Col-0 (Figure 3A). Unexpectedly, *ein2-5* was resistant to ABA. Almost no reduction in chlorophyll content was observed in the 1 μM ABA treatment, and only a slight reduction in chlorophyll content was observed under the 100 μM ABA treatment (Figure 3A). These observations suggest that *ein2-5* is hyposensitive to ABA in leaf senescence, and that activation of ethylene signaling is necessary for efficient induction of leaf senescence by ABA. Consistent with this idea, *ein3-1 eil1-3* was also hyposensitive to ABA in leaf senescence (Supplementary Figure S3).

Next, the crosstalk between ABA and PIF4/PIF5 was investigated. Because PIF4 and PIF5 degrade under light conditions, we examined ABA-induced leaf senescence under dark conditions where PIF4 and PIF5 proteins are stable (Figure 3B). ABA accelerated chlorophyll degradation in Col-0 under dark conditions, and the acceleration of chlorophyll degradation is significantly inhibited in *ein2-5*, consistent with the observation in Figure 3A. Meanwhile, chlorophyll degradation was not delayed in *pif4 pif5* (Figure 3B and Supplementary Figure S4), suggesting that ABA does not promote leaf senescence through the function of PIF4 and PIF5. In contrast to our observations, Zhao et al. (2016) did not observe a delay of ABA-induced senescence in ethylene insensitive mutants. However, one difference is the lines which were used in the experiments. Zhao et al. (2016) used *ein2-1*, which is a leaky allele of *ein2*, and *ein3-1*, which shows complete ethylene insensitivity only when accompanying a mutation of its paralog *EIL1*, whilst, in this study, we used the null allele *ein2-5* and *ein3-1 eil1-3*. We then treated *ein3-1* with ABA, but found that *ein3-1* also shows a delayed senescence phenotype, although it is slightly weaker than *ein3-1 eil1-3* (Supplementary Figure S3). Therefore, the difference of results between our and Zhao et al.’s study could be attributed to the differences in experimental conditions.

### Relationship Between PIF4/PIF5 and Ethylene in Dark-Induced Senescence

**FIGURE 4:**
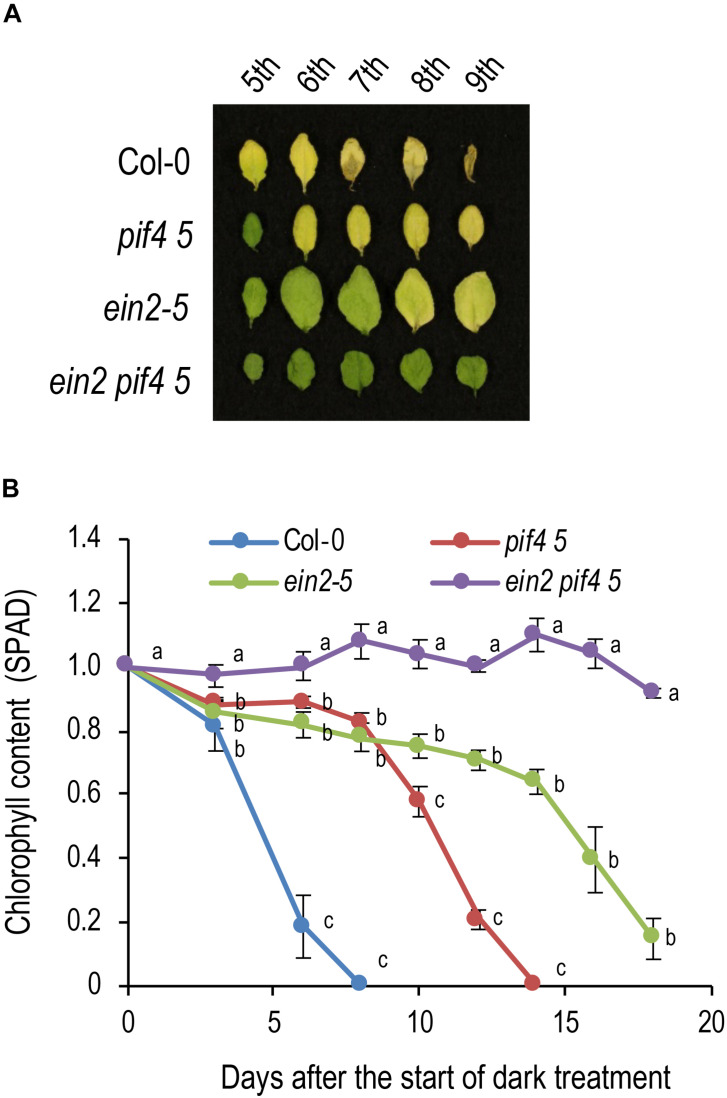
Genetic interaction between *EIN2* and *PIF4/PIF5* in dark-induced senescence. **(A)** Delayed leaf senescence phenotype of the *ein2 pif4 pif5* triple mutant. Leaves in the 5th to 9th positions from the top of 4-week-old plants were incubated in the dark for 14 days. **(B)** Changes in chlorophyll content (SPAD value) over time in the *ein2 pif4 pif5* triple mutant during dark incubation. Detached 7th position leaves were used for the analysis. Data for the same day after the start of dark incubation were statistically compared using Tukey’s multiple comparison method (*p* < 0.05). Bars indicate standard errors (*n* = 3).

Next, we investigated the genetic interaction between ethylene signaling and PIF4/PIF5 in dark-induced senescence. *pif4 pif5* shows a delayed senescence phenotype, but the 7th leaf turned completely yellow under dark conditions at 14 DAT (Figures 4A,B). *ein2-5* showed a stronger delayed senescence phenotype, with the 7th leaf remaining green at 14 DAT and tuning yellow at 18 DAT. The *ein2-5 pif4 pif5* triple mutant had an even stronger delayed senescence phenotype, retaining a high chlorophyll content even at 18 DAT (Figure 4B). The *ein2-5 pif4 pif5* triple mutant also retained relatively high *Fv/Fm* values and low *SGR1* expression even at the stage that *ein2-5* turned yellow, confirming that *ein2-5 pif4 pif5* is a very strong delayed senescence mutant (Supplementary Figure S5). These observations suggest that ethylene has a major role in dark-induced senescence and that PIF4/PIF5 also promotes dark-induced senescence through another pathway in addition to the ethylene signaling pathway.

## Discussion

### The Phytochrome-PIF-Ethylene Cascade Is the Major Pathway Regulating Dark-Induced Senescence

There could be two main factors that induce dark-induced leaf senescence. The first is the inactivation of photoreceptors by light depletion. Analyses of *phytochrome b* (*phyb*) and *pif* mutants have suggested that it is the major cause of dark-induced leaf senescence (Sakuraba et al., 2014; Song et al., 2014; Zhang et al., 2015). The second is the starvation of energy (sugar) caused by a repression of photosynthesis. This study suggests that this may contribute minimally to the promotion of dark-induced senescence. Meanwhile, in natural leaf senescence, defects in sugar signaling delays, whilst sugar application promotes, leaf senescence during natural senescence (Moore et al., 2003; Pourtau et al., 2006). Taken together, this suggests that sugar starvation does not promote leaf senescence in dark-induced senescence and sugar accumulation promotes leaf senescence in natural senescence.

While a number of phytohormones are known to be involved in the regulation of leaf senescence, the degree of contribution of each phytohormone varies. The observations indicating that ABA accumulates during dark-induced leaf senescence (Yang et al., 2014), and that the addition of ABA and overexpression of the ABA receptors PYL8 and PYL9 promote leaf senescence (Lee et al., 2015; Takasaki et al., 2015; Zhao et al., 2016) suggest that ABA is involved in dark-induced leaf senescence. In this study, the core ABA-signaling mutant *srk2d/e/i*, which showed ABA insensitivity in ABA-induced senescence, exhibited a delay of dark-induced senescence, confirming that ABA is involved in dark-induced senescence. However, the strength of repression of leaf senescence in *srk2d/e/i* is apparently weaker than that of *ein2-5*. Woo et al. (2001) and Ueda and Kusaba (2015) demonstrated that strigolactone synthesis and signaling mutants exhibit delayed senescence phenotypes, but the degree is also smaller than that of *ein2-5*. These observations suggest that ethylene is the central phytohormone regulating leaf senescence during dark incubation.

In this study, we also analyzed the genetic interaction of PIF4/PIF5 and ethylene signaling. The *ein2 pif4 pif5* triple mutant showed a stronger delayed leaf-senescence phenotype than *ein2*, suggesting that PIF4/PIF5 could promote leaf senescence independent of ethylene signaling although ethylene is the major regulating factor of dark-induced senescence. In fact, it is known that PIF5 directly regulates expression of the senescence regulating transcription factor gene *ORE1* and chlorophyll-degrading enzyme genes such as *SGR* and *NYC1* (Sakuraba et al., 2014; Song et al., 2014; Zhang et al., 2015).

Meanwhile, *ein2 pif4 pif5* showed a stronger delayed senescence phenotype than *pif4 pif5*, suggesting that ethylene signaling could be activated by another signaling pathway instead of PIF4/PIF5 in dark-induced senescence. It is possible that another member of the PIF family or non-red light regulates ethylene signaling in dark-induced senescence. For example, the blue-light receptor cryptochrome (CRY) and its downstream transcription factor cryptochrome-interacting basic-helix-loop-helix (CIB) are reportedly involved in leaf senescence in soybean (Meng et al., 2013). Although the effect of blue light on dark-induced senescence was not directly examined in the study, this could be an interesting potential avenue of research in *A. thaliana*.

### Crosstalk Between ABA and Ethylene in Dark-Induced Senescence

*ein2-5* and *ein3 eil1* are hyposensitive to ABA in leaf senescence, suggesting that ABA promotes leaf senescence through ethylene signaling. However, leaf senescence induced by ABA progressed in *ein2-5* to some extent, suggesting the presence of an ethylene-independent pathway. *pif4 pif5* did not show hyposensitivity to ABA in leaf senescence, suggesting that ABA does not promote leaf senescence through the function of PIF4 and PIF5. PIF4 and PIF5 may act upstream of ABA in leaf senescence as previously suggested by Sakuraba et al. (2014). Thus, ABA promotes dark-induced senescence in concert with ethylene, though it has less impact on dark-induced senescence than ethylene. Furthermore, the senescence-induced NAC transcription factor AtNAP promotes ABA synthesis via regulation of the expression of *ABSCISIC ALDEHYDE OXIDASE3* (*AAO3*), suggesting a complex regulation of ABA signaling during dark-induced senescence (Yang et al., 2014).

### Roles of Senescence-Induced Senescence Regulators

Genes induced during senescence are called senescence-associated genes (SAGs) (Weaver et al., 1998). SAGs can be classified into two classes, SIEs and SIRs. SIEs include chlorophyll degradation enzymes such as SGR and NYC1 and proteases such as SAG12, which directly cause senescence syndromes. SIRs include NAC transcription factors such as ORE1, AtNAP and WRKY transcription factors such as WRKY53. SIRs are induced along with the progression of leaf senescence and regulate the progression of leaf senescence. Strigolactone synthesis enzyme genes *MAX3* and *MAX4* (Ueda and Kusaba, 2015), ethylene synthesis genes encoding 1-aminocyclopropane-1-carboxylic acid synthase (ACS) (Qiu et al., 2015), and ABA synthesis genes *AAO3* (Yang et al., 2014) might be included in the SIRs group, in the context that they could regulate leaf senescence.

Among transcription factors that regulate leaf senescence, PIF4 and PIF5 are not included in the SIRs as the stabilization of PIF4 and PIF5 proteins is due to deprivation of light and is not associated with senescence. Genes involved in shade avoidance are known to be under the control of negative feedback regulation to avoid excess signaling (Roig-Villanova et al., 2007; Hornitschek et al., 2009). We revealed that the expression of the shade avoidance genes *HFR1*, *PIL1*, and *AtHB2*, which are directly regulated by PIF5, was upregulated at 1 DAT, but downregulated at 2 DAT and later on during dark incubation. This observation suggests that the expression of these genes is also under the control of negative feedback, which is mediated by hetero-dimerization between PIF5 and HFR1, in the detached leaves during dark incubation. This means that the transcription activation activity of PIF4/PIF5 is limited at 1–2 DAT during dark incubation. In contrast, the expression of senescence-inducible genes *SGR1* and *ORE1*, that are also direct targets of PIF4/PIF5, continues to increase at 2 DAT and later, which appears to inconsistent the expression pattern of *HFR1*, *PIL1* and *AtHB2*.

This contradiction might be explained by the hypothesis that, in addition to PIF4 and PIF5, another factor(s) regulates the expression of *SGR1* and *ORE1*. The stabilized PIF4 and PIF5 enhance the transcription of *PIL1*, *AtHB2*, and *HFR1* and *SGR1* and *ORE1* in the early stage of dark-induced senescence. In the later stage, transcriptional activity of PIF4 and PIF5 are downregulated by the negative feedback regulation and consequently, *PIL1*, *AtHB2*, and *HFR1* are downregulated. On the contrary, expression of *SGR1* and *ORE1* might be further upregulated by other transcription factors, possibly SIR transcription factors. The observation that *SGR1* and *ORE1* were induced, albite slightly, on the first day of dark incubation is consistent with the notion that PIF4 and PIF5 regulate these genes in the early stage.

Liebsch and Keech (2016) present a feedforward loop model of dark-induced senescence. They hypothesize that PIFs play a major role in initial light deprivation, that EIN3 in addition to PIFs enhances the expression of senescence-related genes in “prolonged light deprivation,” and that ORE1 further promotes senescence in “persistent darkness.” Our observations are largely consistent with their hypothesis. PIF4 and PIF5 stabilized by dark incubation induce the expression of SIR transcription factors and some SIEs in the early stage of dark-induced senescence. In the later stages, SIR transcription factors may mainly function in promotion of leaf senescence.

The majority of SIRs represented by ORE1 and AtNAP are positive regulators of leaf senescence. However, some SIRs such as VNI2 and JUB1 are negative regulators of leaf senescence that are induced during the senescence (Yang et al., 2011; Wu et al., 2012). Although each SIR has a specific function, leaf senescence may be regulated by “total activity” of SIRs to enhance SIE function and cause senescence syndrome. Such total activity of SIRs could be referred to as senescence signaling (Ueda and Kusaba, 2015).

### A System Eliminating External Noises That Induce Accidental Leaf Senescence

**FIGURE 5:**
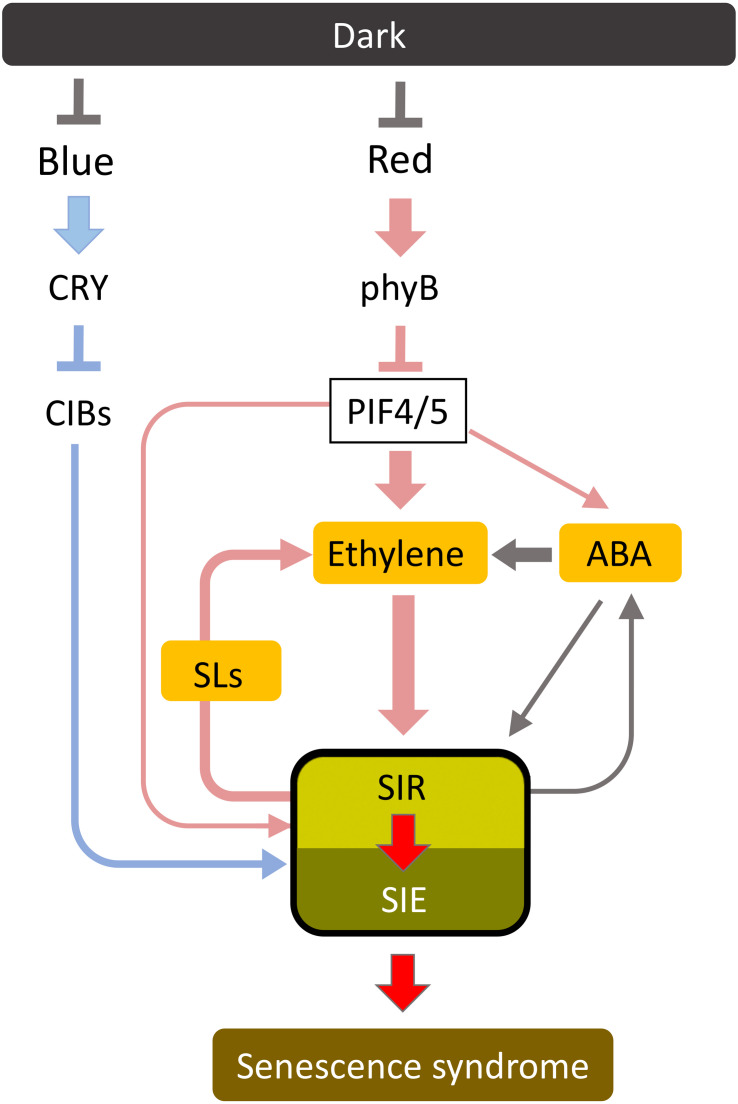
A proposed signal transduction pathway of dark-induced senescence. Dark-induced senescence is mainly regulated by the red light pathway involving phyB and PIF4/PIF5. PIF4 and PIF5 regulate ethylene synthesis and signaling to progress leaf senescence in addition to ABA signaling. ABA mainly promotes leaf senescence via ethylene signaling. PIF4 and PIF5 also directly regulate senescence-induced senescence regulators (SIRs) including the NAC transcription factor ORE1, and senescence-induced senescence executors (SIEs) such as Chl*a*-degrading enzyme SGR1, at the early stage of leaf senescence. At later stages, SIRs enhance their own expression and activity through a positive feedback regulation, which elevates SIE expression and reinforce leaf senescence (senescence syndrome). Furthermore, blue light is thought to repress dark-induced senescence through cryptochrome (CRY) and cryptochrome-interacting basic-helix-loop-helix (CIB).

Leaf senescence can act as a suicide signal, and therefore must be strictly regulated in plants. Plants are exposed daily to external noises that could induce senescence, such as subtle stresses that induce ethylene production and diurnal short-period dark conditions. Such noises must be eliminated to avoid unnecessary premature senescence and plant death. In addition, leaf senescence should progress slowly enough for the plant to salvage nutrients from senescing leaves. Such conditions may be achieved via a multi-step activation system. Figure 5 shows a model of the signal transduction pathway of dark-induced senescence. Dark-stabilized PIFs may mainly function in the early stage of dark-induced senescence. PIFs upregulate SIRs mainly through the function of several phytohormones. SIRs may promote leaf senescence through the induction of SIEs. The initial input of senescence-inducing stimuli may not work effectively, which eliminates external noises, while continuous input leads to the activation of SIRs and the steady progress of leaf senescence. Inhibitory SIRs such as VNI2 may also play a role in its fine tuning. Thus, the multi-step activation system may provide both a kind of robustness for growth and efficient nutrient salvage when required.

## Data AvaIlability Statement

All datasets generated and analyzed for this study are included in the article/Supplementary Material.

## Author Contributions

MK and HU designed the study. HU, RI, YM, TI, and YN conducted the research. MK, HU, and TK wrote the manuscript.

## Conflict of Interest

The authors declare that the research was conducted in the absence of any commercial or financial relationships that could be construed as a potential conflict of interest.
