# Supplementary figures and images for: Genetic Interaction Among Phytochrome, Ethylene and Abscisic Acid Signaling During Dark-Induced Senescence in Arabidopsis thaliana
Source: Front Plant Sci. 2020 May 21;11:564. doi: 10.3389/fpls.2020.00564 (PMC7253671; doi:10.3389/fpls.2020.00564)

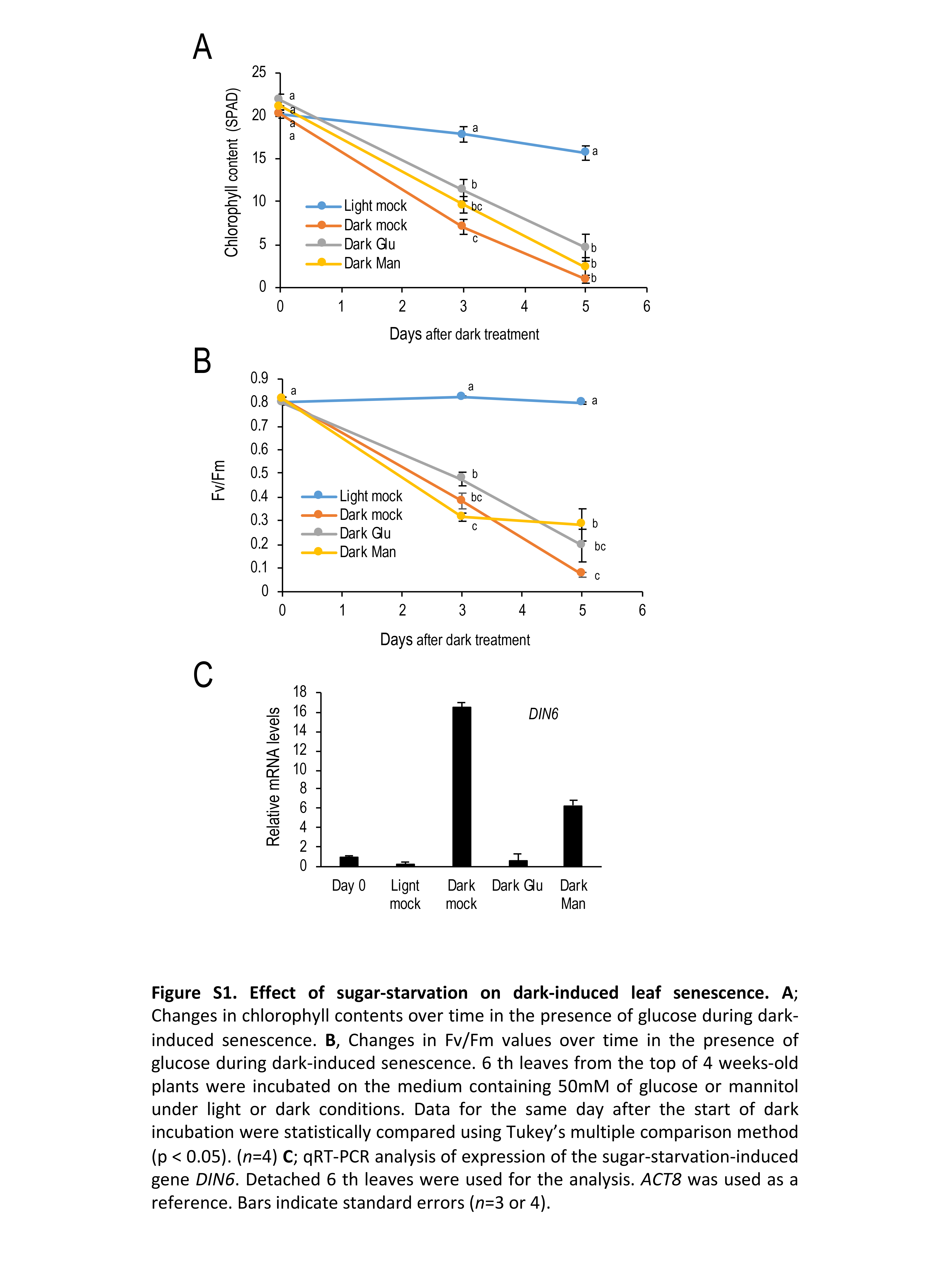

Supplement: Supplementary file 1 [file Data_Sheet_1.zip › Image 1.jpg]

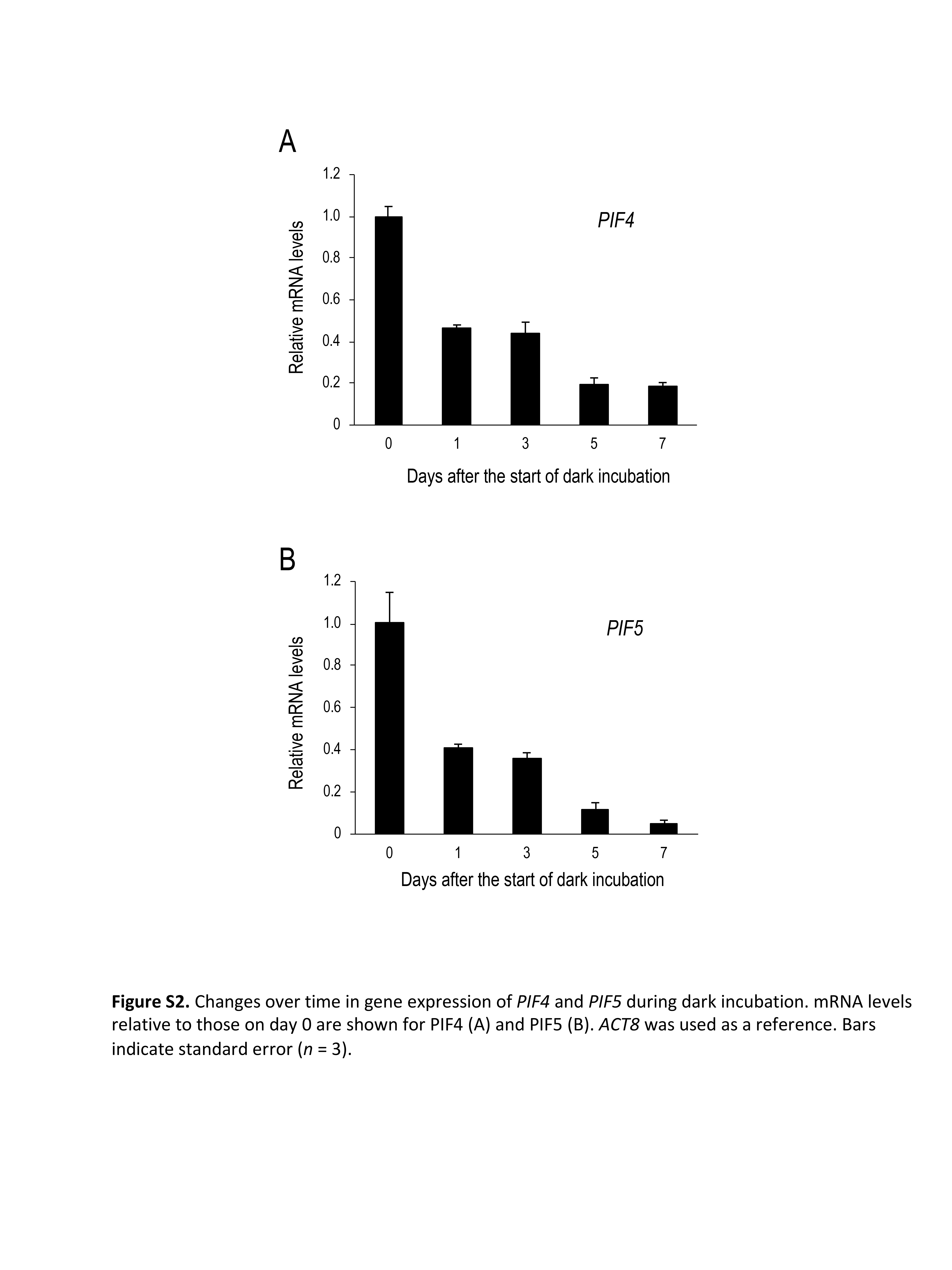

Supplement: Supplementary file 1 [file Data_Sheet_1.zip › Image 2.jpg]

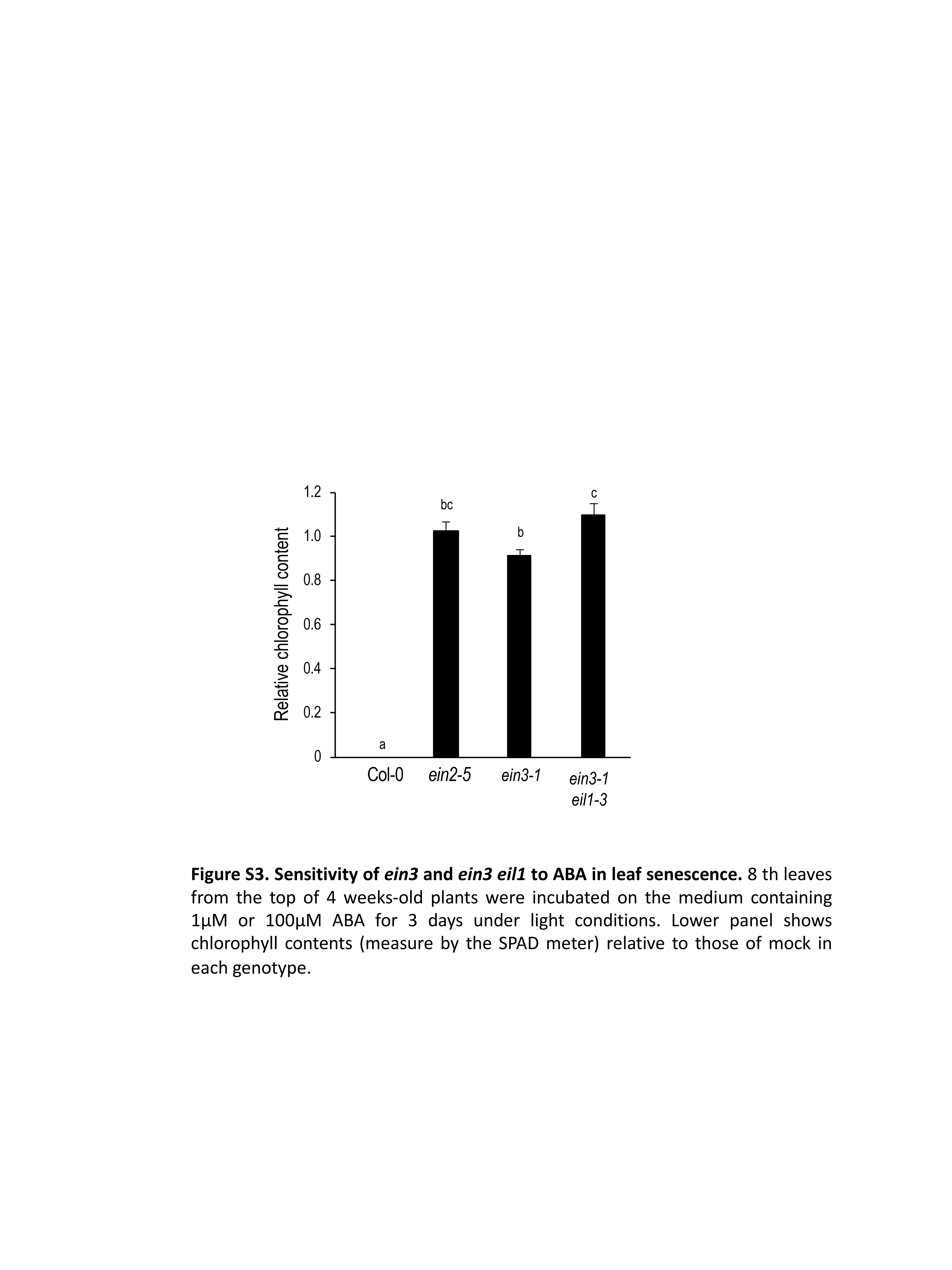

Supplement: Supplementary file 1 [file Data_Sheet_1.zip › Image 3.jpg]

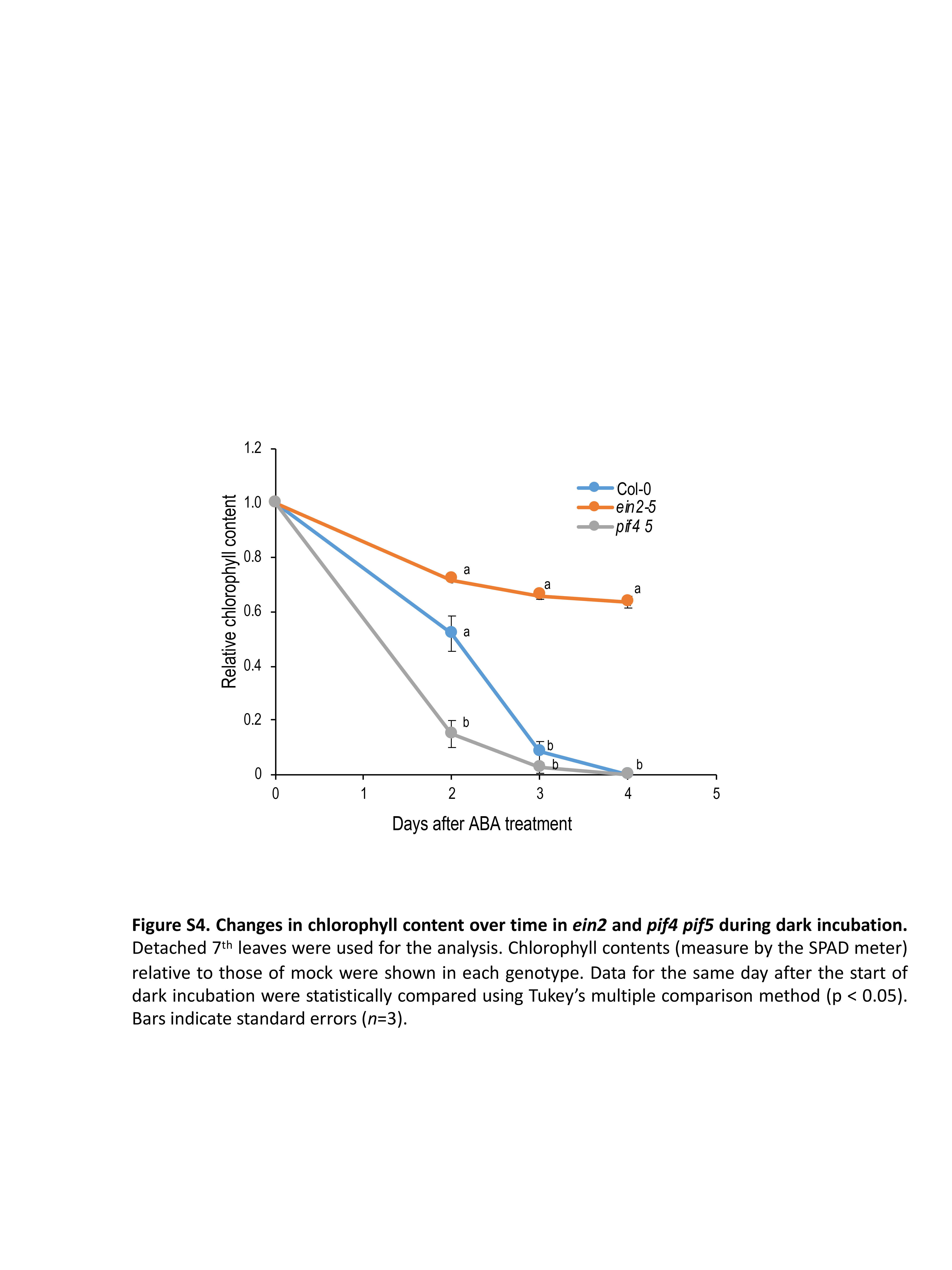

Supplement: Supplementary file 1 [file Data_Sheet_1.zip › Image 4.jpg]

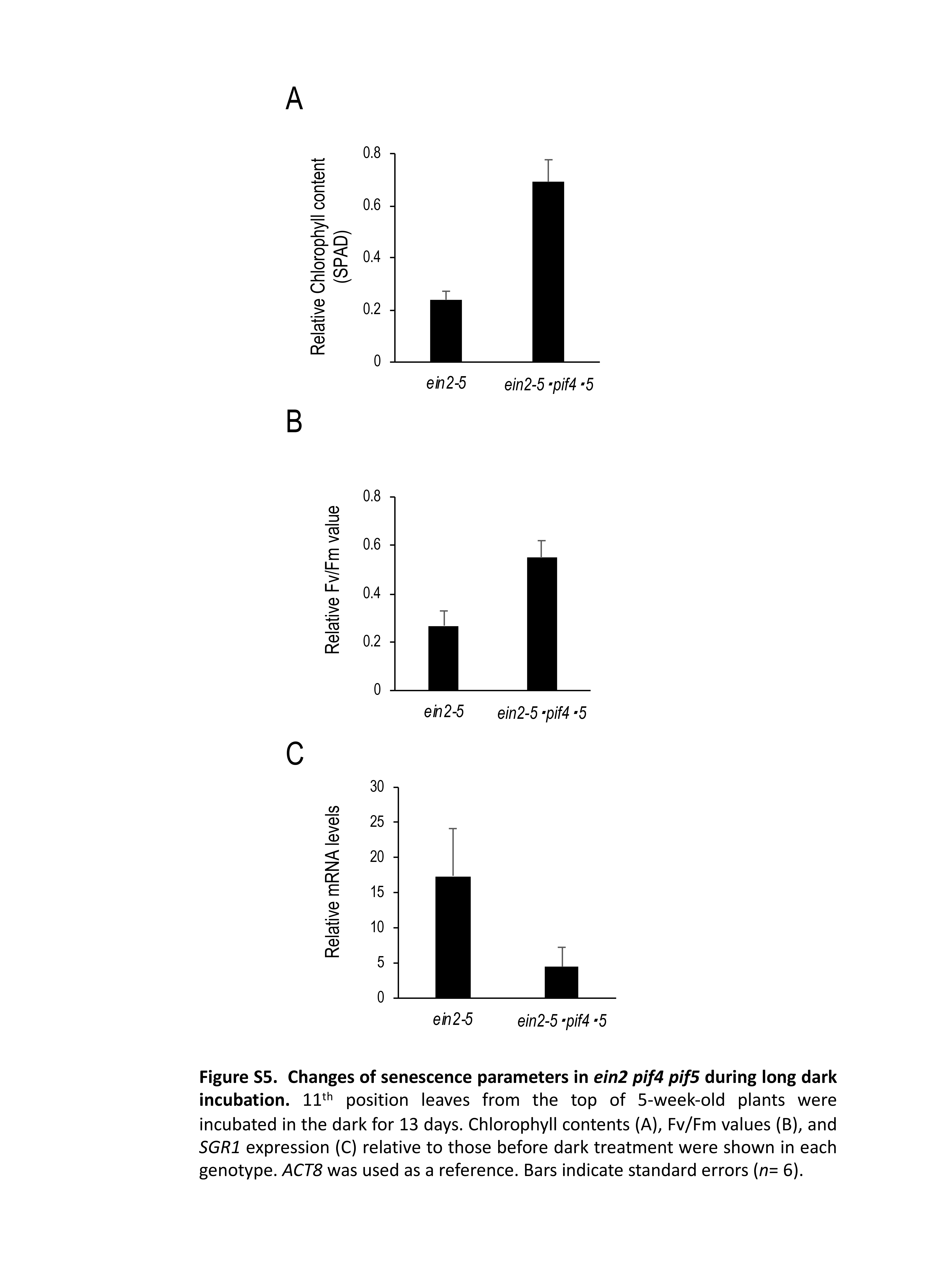

Supplement: Supplementary file 1 [file Data_Sheet_1.zip › Image 5.jpg]

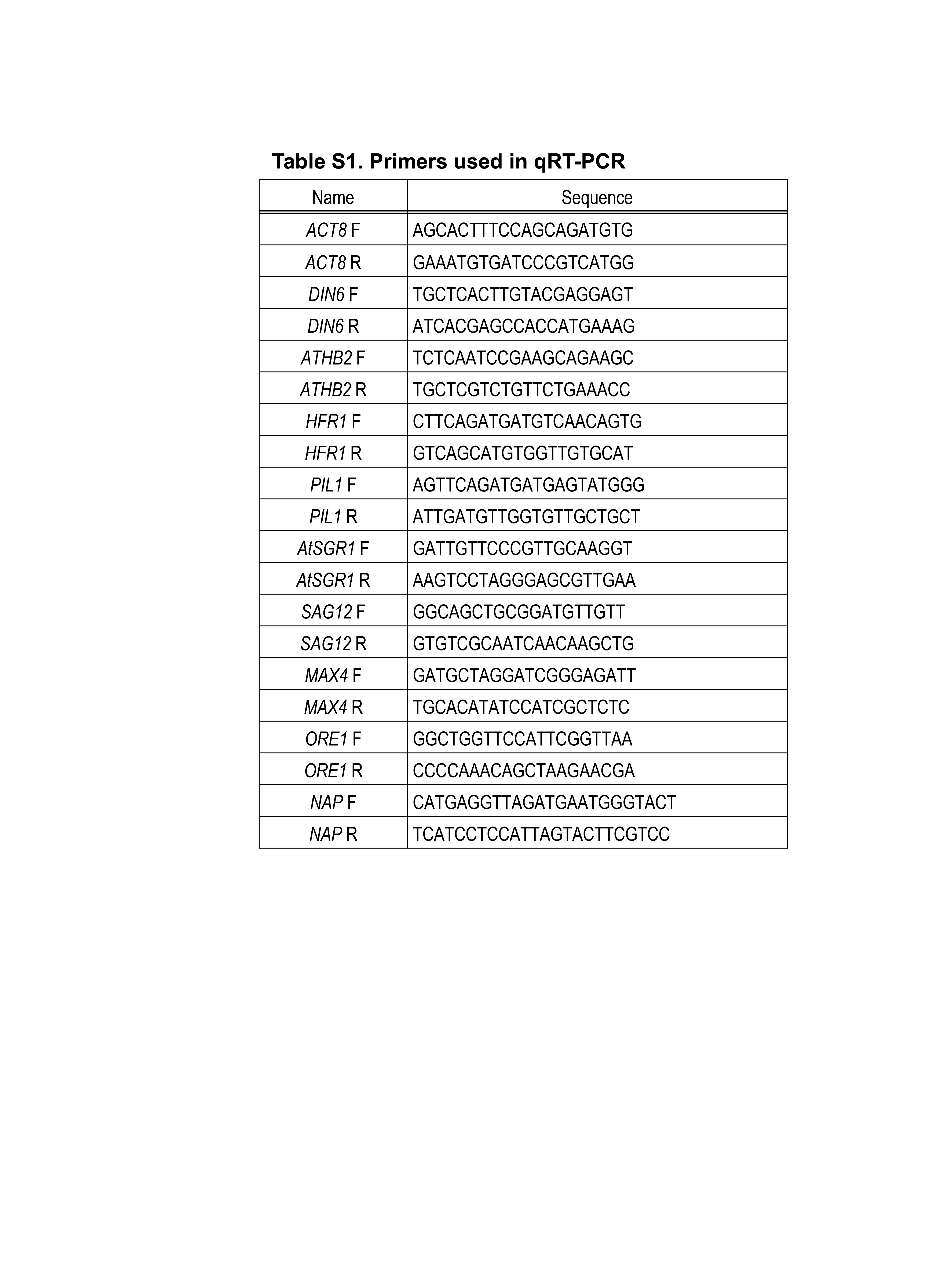

Supplement: Supplementary file 1 [file Data_Sheet_1.zip › Table 1.jpg]
